# Illinois State Dental Society

**Published:** 1886-08

**Authors:** 


### ILLINOIS STATE DENTAL SOCIETY.

REPORTED EXPRESSLY FOR THE INDEPENDENT PRACTITIONER.

Continued from page 372.

#### THURSDAY AFTERNOON SESSION.

Dr. Cushing, chairman of the committe on memorials, presented a sketch of the life and service of Dr. Isaiah Forbes, of St. Louis, one of the oldest of the honorary members of the society. Resolutions of sympathy with surviving friends were unanimously passed. Speeches of eulogy were made by Drs. Sturgiss, Black, McKellops and Taylor.

The subject of Operative Dentistry was again called up.

*Dr. Green*—Believes that one cannot properly fill a tooth without full command of space. Many of the old fillings inserted twenty years ago are still in good condition, and he feared that a smaller proportion of the fillings of to-day will be as perfect twenty years hence. The old fillings were usually inserted with a large amount of space around them.

*Dr. Brophy*—Has been surprised to see operators in the clinics attempt to fill teeth around a corner—to condense gold with a cork-screw. The only way in which a cavity can be successfully filled is to get positive and direct access to it. He has no faith in guess work. If he cannot see every part of the cavity, he does not fill. We do not, as a rule, polish the margins of the cavities sufficiently. When little projections and inequalities are left they are fractured, and the work thus unduly exposed. Small and fine stones should be used to polish the edges of cavities, and sufficient space should be gained to enable one to do this. Some new burs have been introduced, in coarseness between gold finishing and the regular cutting burs, and these are very useful. A filling should have its final examination under a high magnifying power, when all the imperfections, if any exist, will be exaggerated and recognized. We hear methods discussed and know that some of them are excellent. Then we go home and think of them no more. Dr. Black once demonstrated how cohesive gold could be made non-cohesive. Much of the gold that is sold for soft is not so. It will not bear wedging, and will bridge over a cavity. We can easily make gold non-cohesive by exposing it to the fumes of ammonia.

*Dr. Townsend*—Sometimes a cavity extends beyond the gum, and it is necessary to see the cervical wall. This can only be attained by prolonged and very gradual wedging. Perfect contour cannot be obtained without patient wedging.

*Dr. Stevens*—I have never yet seen a tooth in which the pulp had been exposed and capped that was not dead. I know that many dentists are now capping pulps which never were exposed, and reporting successful operations. Capping to protect from thermal changes is not pulp-capping at all. Dentists are altogether too loose in their statement of cases. I have no faith in the transplanting of teeth. In extracting teeth I have seen the adjoining one loosened, but I do not believe that when the relations existing between tooth and jaw are once completely severed, they ever can again become normal. In filling teeth I do not put on the rubber dam until the rough work is done, and the excavation is at least partially made.

*Dr. Dorn*—A young man once came to me who had fallen on the ice and broken off both central incisors, the pulp of one being exposed largely, the other less so. I touched the exposed portions with a solution of nitrate of silver, and capped them with Dawson's mineral cement; six weeks later I removed a part of the cement. leaving enough for a cap. Both pulps appeared to be alive, and I completed the operation with contour gold fillings. Two years subsequently the pulps were alive, or appeared to be so.

*Dr. Davis*—It frequently takes considerable time to reveal the true status of such cases.

*Dr. Sitherwood*—Said that one of the most useful of the late devices that he had seen was the invention of Dr. Geo. L. Field, of Detroit. A lens, three or four inches in diameter, is by means of a series of universal joints attached to the chair in such a way that when the patient's head is in position it may readily be brought into proper focus over the teeth. The focal distance is such that it is quite out of the way of the patient, while it is between the eye of the operator and his work. It throws a flood of light into the mouth, besides magnifying the work so that any imperfection may be readily seen.

*Dr. Barrett*—Had seen the same device, and desired most strongly to commend it. To those who had reached middle life, and who began to feel the necessity for magnifying glasses, he thought this would prove a great blessing. Dentists abuse their eyes. Their work is very trying, and they do not use eye-helps as much as they should. The eyesight would be longer preserved if they employed magnifiers, and thus avoided unnecessary strain upon their vision. Besides, work is frequently imperfect because of microscopic defects, which only a glass will reveal. The magnifier of Dr. Field is very convenient, and may be swung into place and out as readily as the bracket table. It will serve all the uses of spectacles for old dentists, and magnifiers for young ones, while it has the advantage of never getting lost.

*Dr. Stevens*—I am astonished at the wonderful acuteness of vision of many dentists who speak of seeing the dental nerve without any magnifiers. It is something I was never yet able to do, in my life. I have seen exposed dental pulps, but never a dental nerve.

*Dr. Black*—Said that the normal function of the tooth pulp is not one of touch. There is no localizing sense, but the nerve simply conveys a sensation of pain alone. The main office is to give notice of thermal changes. When the tooth-pulp is excited, these alterations in temperature are exceedingly painful, and a hyperæmic condition is the result. The continued thermal shocks aggravate this, until there is such congestion that stasis of the blood current and death of the pulp results. To protect the tooth from these shocks he warms common red gutta-percha, and with the fingers moulds it about the affected tooth and the adjoining one. When cold, this is taken from the mouth and properly trimmed, and then replaced. In two cases out of three, by this means, pulps may be saved; at least that is his experience, and hoped that each one would try it and become convinced of its usefulness.

A paper was read by Dr. J. D. Moody, of Mendota, upon

### POST GRADUATE STUDY.

A college training, said the essayist, is excellent, but the principal part of a man's education is that which he gains for himself, unaided by the schools. There are those who are longing for something higher, better, wider than that which professional colleges give us, and to such a few suggestions will not be out of place. The medical training of the future is to be hygienic and sanitary, rather than medicinal. It may be so in dentistry, but before this there must be a change in some of our methods of instruction. Have we, as dentists, laid the necessary foundation structure for a true scientific course of study? Two years is not enough for this. Clergymen spend four years in college and three years in a theological seminary. Medical men require quite as much for professional study, and can a dentist worthy of the name be made any quicker? A true student must have something more than mere desire for the acquisition of strictly professional knowledge. He must be prepared to investigate scientific fields for himself, and not always to receive his knowledge at second-hand.

There are few men who can do good and original work before they are forty years of age. Previous to that the judgment is not sufficiently matured, the experience is too limited, and the faculties of observation are not enough trained. Our part of the field of science is constantly widening, yet careful reading of our journals shows that but few new names have come to the front within the last ten years. The growth and multiplication of the lower forms of life offers one of the very best fields for study conceivable. Let us look for a moment at the literature of this branch of science. No name shines with a brighter lustre than that of Dr. W. D. Miller. If I take up a volume of the INDEPENDENT PRACTITIONER and read one of his articles, I see at once, that to properly comprehend them something besides mere mechanical training is demanded. I must have a degree of familiarity with the fundamental truths of science, and I must be fully up with the latest medical facts and theories. If I read Dr. Black's book on "Poisoning by Micro-Organisms," I must, for its understanding, have some previous training, and a knowledge of the basal facts upon which it is founded. If I read Dr. Barrett's articles on "Nervous Force," I shall not be edified if I do not comprehend some of the principles which govern the correlation of forces, and have some knowledge of what force is. If I take up Dr. Abbott's and Dr. Bödecker's studies of the pathology of the tooth, I must have some previous knowledge of tooth structure or I shall obtain little benefit from them. It is the same with all technical writings. It needs a general knowledge of physics to enable one to comprehend papers on any philosophical subject. If a man makes the best use of his time, the minutes spent between twenty-five, the age of graduation, and forty, the year of best development, will thoroughly prepare him for original investigation in almost any field.

In the consideration of this subject, and to aid in the preparation of this paper, the author had corresponded with a number of men prominent in dentistry, and had asked what, in their opinion, would be a proper subsequent course of study for those who had graduated at a dental or medical college. The answer had been crystallized into the following scheme, which might be pursued in such order as the student found most practicable. It does not pretend to be perfect, or entirely comprehensive, but it will at least be suggestive:

1. Physics: Molecular and Mechanical.
2. Electricity and Magnetism.
3. Chemistry: Organic and Inorganic, with Chemical Physics.
4. Metallurgy.
5. Microscopy: Technical only.
6. Zoology: Invertebrate Morphology.
7. Botany: Systematic.
8. Anatomy: Human and Comparative.
9. Embryology.
10. Odontology: Comparative.
11. Histology: Vegetable and Animal.
12. Physiology: Vegetable and Animal, with Chemical Physiology.
13. Bacteriology.
14. Pathology: General and Dental.

A working knowledge of these branches could not be obtained in less than fifteen years; so at forty a man should have a good practical knowledge of these subjects, and at that age he would be prepared for earnest work. Unless we comprehend basal principles, we are not prepared to practice any profession other than empirically. We must make study attractive. Our work should appeal to the intelligence rather than to the sordid side of our natures.

How shall such a course of study be carried out? A special school with a higher degree would require five years of special study, and this few can afford. The lack of a plan has been felt and expressed in many ways. To meet such a want this paper has been prepared. The most practical plan and that which promises the most advantage, would seem to be a post-graduate correspondence school, which would not demand that students should leave their homes. Competent instructors should be designated and new books must be prepared, for we have not the proper text-books. The work would be guided by correspondence with the tutors, only one or two studies being pursued at once and the time unlimited. Then, upon the completion of the course, a degree should be conferred, and for this he would suggest D. D. Sc., or Doctor of Dental Science.

Who shall organize such a course? Our best educators. The full benefits would mostly accrue to the young graduate, but all would reap advantages.

The discussion was opened by Dr. C. N. Johnson, who said: The chief objection to such a scheme is that the members of our profession have not a sufficient general education. To properly study such technical subjects requires some knowledge of ancient languages, as the nomenclature of science is mostly derived from the Latin and the Greek. Very few of our men are classical scholars. They have not the mental training for such work. We are not old enough in scientific knowledge and culture. If a special degree is to be instituted, we need one that is not so near like the present dental degree. A scientific and not a professional diploma should be given. The list of studies is a good one, and the arrangement is excellent. The placing of the study of Vegetable Physiology before that of Animal is especially to be commended. The most of us have more time than we think we have. Those who really should have the most leisure are the ones who complain of lack of time, because they have not learned systematically to economize their moments. We do not usually operate more than eight hours. One hour for study each day would not be much, but in a week this will give us a very good day, and in a year it proves a great aggregate. In a lifetime, one hour's judicious study each day will make of almost anyone a scientist. Adjourned.

THURSDAY EVENING SESSION.

After the transaction of the usual routine business, Dr. J. G. Templeton, of Pittsburgh, as a delegate from the Pennsylvania State Dental Society, presented the fraternal greeting of his brethren, and extended an invitation to the members to attend the next annual meeting at Cresson.

Dr. Brophy moved that Dr. Harlan be invited to present before the next meeting the new remedies described in his paper, and such old ones as he may deem best, that members may become familiar with them.

Drs. Brophy, Swain, Kitchen, Marriner and Cushing were appointed a committee to take into consideration the devising of some way to render Dr. Black assistance in his investigations, and to enable him to devote his entire time to that work.

Dr. Black made his regular report, as follows :

Last evening I made a number of plants in illustration, and I have one that seems to be pure. Four other tubes are sterile. The one which succeeded is *Streptococcus magnus*, which we find so abundant under artificial plates. I planted a number of gelatogens, or gelatine-forming bacilli. You see that I can turn this tube, the bottom side up. A cap of gelatine has been formed that makes it appear as if it were all solid. In a week or so the gelatine will become red, and in a short time longer this will liquefy and be filled with round spores. Those gelatine-forming bacilli give to the teeth a gummy appearance.

Here is a tube of caries fungus, planted three days ago, and to make the illustration complete, we will take a tube of control matter and mark the difference. You will see that the control is neutral, while the infected solution is strongly acid. As a further illustration that this acid is formed by caries fungus, I will show a plant made yesterday by passing the sterilized wire between the lips of a young lady. You note the difference. My principal object in making these cultivations before you is to render you familiar with some of the foundation facts in oral bacteriology and pathology.

The discussion of Dr. Moody's paper on Post Graduate Study was then declared in order.

*Dr. Barrett*—Said that it was very seldom that a society was privileged to listen to so thoughtful a paper as was this. It was farseeing, for it looked to the future of our profession. If we are to be indeed a scientific body, we must not always be engaged in the study of applied science. We may become skillful mechanicians and accomplished operators, but that will not make of us scientific men. A physician may be a thorough physiologist and anatomist, well versed in materia medica and pathology, an acute diagnostician and an accomplished practitioner, but all that will not give him standing in the world of general science. He must have made some advance in abstract study. He must have investigated pure science and completed some original investigations if he would be known among scientists. We are good *dentists,* but that does not necessarily mean that we are philosophers. This cry of "no time" for reading and study does not come from the leading men in dentistry. Look about you and see who are the men of the greatest general labors—men who occupy leading positions and who do the most for their profession, men of culture, who read all the dental journals and books, and you will find these are the men with the largest practice. There is leisure enough in dentistry, but is there the disposition for study? Is there the general enlightenment and classical training to make such a course as this practicable? That remains to be seen. At any rate, the effort to establish such a course is highly commendable, and ought to succeed, whether it does or not.

*Dr. Black*—I was much interested in this paper. It is not necessary for me to commend it. I wish to emphasize another point. Few men have any just appreciation of the amount that may be accomplished if one hour of each day is devoted to systematic study. It will be sufficient of itself to make a wise man. It will develop the mental faculties and resources to a degree that will surprise anyone. Whether or not you choose to follow this or any other particular scheme, give one hour each day to regular systematic study, and when you have become interested you will give more.

*Dr. Sitherwood*—I have listened to no paper that has so interested me, and hope that this subject will not be dropped, but that a committee will be appointed to devise something akin to the Chautauqua Literary Course.

*Dr. Harlan*—Like the other members, I listened to Dr. Moody's paper with great interest. Most young graduates do not appreciate the necessity for further study until they find themselves in the rear of their profession. Until recently there were no facilities for post graduate study, but now there are institutions where one may pursue any desired plan of investigation. Every man should have some special field in which he shall be constantly engaged in study, and if it be only the collection of curious epitaphs, it is better than no study at all.

*Dr. Kitchen*—If members of this society had never studied anything but red-rubber and amalgam, they would not have made the progress they have. I hope that we shall, as a society, engage in some specific plan of original investigation.

*Dr. Brophy*—The National Association of Dental Colleges has appointed a committee to take into consideration the supplying of the profession with a complete set of text-books.

*Dr. Barrett*—That would seem to imply that it was only from the ranks of the teachers that we were to expect books, while the history of the literature of the world teaches us that the best books come from the most unexpected sources. It is very seldom that a great book is the result of the definite appointment to the task of even the man whom all the world agrees is most competent to write upon any special subject.

*Dr. Moody*—In closing the debate, said that this subject of scientific study had been near his heart for a long time. He deprecated any hasty action. The work should be a national one, and he had brought the subject up that it might be duly considered. It is true that we have not the training now to do real scientific work, but we must educate ourselves. We have not to-day a properly prepared dental text-book. In anatomy we have only Gray, a book of many hundred pages, and it is absurd to put an untrained mind to the study of such a work as this. We have not the basal principles tersely laid down in dental books, and one of our greatest needs is better text-books.

A paper was read by Dr. J. G. Reid upon

### ORAL CHEMISTRY.

Instead of sharing the general progress of dental science, said the essayist, dental chemistry seems to be advancing rearward. This seems to arise from a lack of interest in the subject and because other fields are more inviting. Attention is directed from it by the ascribing to physiological and pathological changes those which should be studied in connection with chemistry. Yet the microscopist needs the aid of the chemist, and there is a vast field opened for the labor of the dentist in the studying of the condition of the fluids of the oral cavity.

The saliva is the principal fluid of the mouth, and that is incapable of exercising any deleterious influence upon the teeth. In its normal condition it is either entirely neutral or possessed of a slight alkaline reaction. If it be found acid, it is an indication that it has undergone some chemical change, not through its normal constituents, but because of the introduction of something from outside. During childhood and youth its condition is usually constant, and its chemical state neutral, or nearly so. There has never been a thorough chemical analysis made of the fluids of the mouth in diseased conditions to ascertain exactly what acids are present. Miller determined lactic acid, but previously there was no knowledge of what the acids were. When we come to examine the process of fermentation and the result of this process, we can scarcely refrain from exclaiming "Eureka."

The chemists of the sixteenth century were the first to notice fermentation. At the present day the physiological chemists are minutely examining this mysterious action, and they find therein the key to much that had previously been unaccountable. Observers have recorded that they found albuminoid matter in great quantities in the saliva as found in the mouth, and the saliva was acid, but they do not tell what was the acid found. An examination into all the conditions will convince us that in most cases this is undoubtedly lactic acid. (The essayist reviewed the conditions under which these acid changes must take place, and showed that it may be, and usually is, lactic acid.)

Lactic acid fermentation is much more active in the carbohydrates, because it is produced at the expense of the sugar. The sugar that is produced from amylaceous substances by the ptyaline of the saliva, is probably in a more favorable condition for lactic fermentation than that which may be introduced from without. This fermentation, it might naturally be supposed, would be more active in the sheltered pockets between the teeth, for here the food may remain for some time undisturbed. The sum of the whole is this:

1st. Dental caries is more active during childhood and youth, as the mouth is not kept in as cleanly a condition, and because the oral secretions being in a neutral or alkaline condition, fermentation it more active.

2d. The presence of fermentable products being generally constant, lactic fermentation proceeds uninterruptedly for an indefinite length of time.

3d. Lactic fermentation, as a rule, precedes other fermentations.

4th. The points of fermentation and decay are coincident.

5th. The acids produced are dilute, but being constant their action is finally destructive. Adjourned.

FRIDAY MORNING SESSION.

Dr. Black reported as follows:

I brought my microscopical garden to this place with some misgivings. My object was to make you more familiar with the fungi of the mouth, and that the subject might not seem to you afar off. I wished to convince you that the demonstration of 'these fungi is not a mysterious process, but that it is as simple as any other gardening. It is merely the raising of vegetables. We get poisons and medicines from visible plants, and we may get the same from microscopical ones. In the progress of the work here I have succeeded better than I had anticipated, The air has not been pure and there has been consequent contamination, but I have succeeded reasonably well. I have made forty-one original plants or impregnations. Out of these I have had thirty-seven growths. Nine are practically pure cultures of caries fungi. The other growths have been impure. I have demonstrated to you the acid-producing power of fungi, and the fact that acids cannot be produced without the presence of sugar or starch. The microscopical exhibition has been a disappointment. Not enough of instruments or lenses have been presented, and it will take time to appreciate the importance of this branch of dental science. The usual lenses for histological work will not answer. We need the modern homogeneous immersions. I have had but one such, and the exhibition has therefore been somewhat restricted. Next year I think we shall do better. We hope to learn something during the year, and to grow into an appreciation of the importance of the subject. The results of the growth of most of the fungi are unknown. We have studied but a few. I have spoken very little of their location or habitat. There is a great field open and inviting, in the careful study and tabulation of the results of observations of fungi of the mouth.

*Dr. Davis*—Are these organisms purely vegetable, or animal; and if they are the former, what does Dr. Watt and others mean by speaking of the "bug theory?"

*Dr. Black*—They are vegetable, and the instances referred to are an indication of ignorance, when honestly applied. When not, it is intended to throw ridicule upon scientific research of this kind.

Discussion of Dr. Reid's paper upon "Oral Chemistry" was declared in order.

*Dr. Black*—This paper is interesting and timely. We have done much work in this field, but we have commenced at the wrong end. The progress in Oral Chemistry has not been commensurate with the labor bestowed. The paper indicates a general progress, and the prospect of better work in the future.

*Dr. Taylor*—There is one point that was not made clear. The author speaks of the acids produced by the fungi, but pays no attention to mineral acids. People often make the remark that they have taken so much medicine that their teeth are ruined. It is not the medicine which is in fault, but because of the cause for taking the medicine—the constitutional condition. It is not usually the acids which are to be blamed, but the general tone of the system.

*Dr. Townsend*—We have been taught that the injury to the teeth during typhoid fever is due to drying of the saliva, and to acid changes.

*Dr. Black*—When, in typhoid fever, the teeth become coated with gelatine-forming fungi, we know that acid formations are active. Alkaline micro-organisms sometimes get control, and then we may not have decay of the teeth, although they are gummed up.

*Dr. Townsend*—Have you ever examined the sordes of fever?

*Dr. Black*—I have not, but it is probably due to micro-organisms.

*Dr. Taylor*—If I understand Dr. Miller aright, all decay is not due to fungi.

*Dr. Black*—It is not possible that mineral acids should produce decay. If they be in sufficient strength to affect the teeth they would destroy the organisms, and caries of the teeth is *never* present without them.

*Dr. Ottofy*—Why is it that with some decay there is an alkaline reaction?

*Dr. Black*—I have never seen such a condition, save where the fungi are dead and decay is stationary—is not progressing.

*Dr. Ottofy*—Is decay never progressing when the mouth is alkaline?

*Dr. Black*—No, sir; most emphatically. In such conditions decay has ceased for the time. In every case where pus is forming and being discharged, decay has ceased.

*Dr. Harlan*—The most important points made in the paper are confirmatory of Dr. Black's opinion, that the lactic ferment which is most powerful in producing the dissolution of the tooth is due to organisms, and that its formation precedes the carious process.

*Dr. Moody*—We are too apt to drop a subject when it has been but partially worked out. I hope that Dr. Reid will not cease his labors, but that next year he will be prepared to give us the results of further study.

*Dr. Kester*—The works upon dental chemistry are few and not recent. This paucity of our literature is due to a lack of demand. Such papers as this will create a desire to know more. Dr. Moody has shown the necessity for study of basal principles, and it would seem that this meeting marks an advance. We have in the mouth all the factors of chemical action—the fermentable substances and the ferments. Here is a great field for study. I fully agree with Dr. Black, that mineral acids play no part in dental caries.

*Dr. Cushing*—I know little of chemistry, but I wish to emphasize the remark that we should encourage the study of this field by men like Dr. Reid, and the Executive Committee should see that the subject is continued. We are upon the threshold of important discoveries. Scientific investigation is but in its infancy, and it is gratifying to know that with such slender encouragement so much work has been done.

Is it a fact that the excessive use of sugar acts disastrously upon the teeth, and if so, is it not because it furnishes food for micro-organism, rather than because it is injurious of itself? I have seen destructive effects from the use of acid confectionery. Are these results also due to micro-fungi? We are constantly asked by parents if candy and confectionery are injurious to the teeth of children. My answer has been that they are not, if the mouth be cleansed after their use.

*Dr. Black*—It seems certain that the indulgence in sweets will vitiate the fluids of the mouth, even though the utmost cleanliness be observed. It will entail a loss of appetite and certain general disturbances. Aside from this, sweets are not injurious. With sour-drops there is a large amount of acid, but I think ill effects are due to constitutional disturbances. Sugar is not injurious to teeth. Magitot placed teeth in a solution of sugar, and at the end of two years they were found perfect. But when fermentation proceeds the teeth are destroyed.

*Dr. Swain*—Is there not the same danger from starch as from sugar?

*Dr. Barrett*—Dr. Black is correct in the scientific aspect of the case. It should be borne in mind that some sugars are fermentable and some are not. One of the best preservatives that we have against putrefaction is cane sugar. With it the housewife prepares her fruits and the butcher his meats. Sugar cured hams are famous the world over. It is not stated what kind of sugar Magitot used to macerate his tooth in, but it must have been cane sugar, and using that he was simply laboring to prove an axiom. Of course it will preserve a tooth, and any school-boy might have told him so. But grape sugar is a fermentable sugar, and this aids tooth destruction. The difference between sugars—cane, grape, milk, and liver sugar—is something incomprehensible. Their chemical formula is the same, and the difference is allotropic. Cane sugar may be converted into grape or vegetable sugar, and then it is fermentable. The organisms of which we have been speaking are capable of converting sugars. Starch, which forms by far the greater part of all our vegetable food, is not digestible as such. It must first be converted into glucose or grape sugar, and this is done with all our amylaceous foods. Cane sugar then, would of itself be preservative of the teeth. When we speak of the presence of sugar in the mouth we do not refer to the usual sweetening of the housewife. Grape sugar or glucose is meant, and this is formed by the conversion of starch, or the farinaceous principles of vegetables. Candy is made largely of glucose or grape sugar, and this part of it would be directly fermentable. It is also more nutritious than cane sugar, though its flavor is not as pleasant.

*Dr. Cushing*—I wish to know if any sugar can be directly injurious.

*Dr. Black*—Sugar is not injurious without organisms.

*Dr. Barrett*—The point that I desire to make is that cane sugar cannot be more injurious than starch. The process of conversion of each into a fermentable sugar is essentially the same.

*Dr. Noyes*—It is time that we began to differentiate concerning decay. There is destruction by more than one method. Acids are formed by cells of mucus membrane as well as by organisms, but until the caries fungi take possession the carious process does not really begin.

*Dr. Reid*—I appreciate the importance of this subject, and am gratified at the attention which it has received. I will endeavor, if possible, to continue the study.

Dr. Noyes moved that the subject of Oral Surgery be taken up, and as the essayist was absent that Dr. Brophy be invited to open the discussion.

*Dr. Brophy*—Many remarks made here would lead to the impression that this, too, is a subject that should receive more attention at our hands. Dr. McKellops has referred to *incurable* alveolar abscesses. Why are they so? It is because of destruction of the alveolus and the deposit of seruminal matter about the root. What is the method of cure? Uusually extraction, but any operator should be competent to treat those cases without resorting to that. Cut out the diseased tissues and the carious bone, which is always present. There is a cavity at the end of the root, and this is filled with some form of deposit. Dr. Black has shown cases in the past in which the alveolar plate of one entire side had been removed, and yet a new one had formed. I have removed everything from the lateral to the second molar, and yet under careful treatment the whole was restored, and the teeth returned to usefulness.

*Dr. Ames*—In the case of an important lateral incisor, like that spoken of by Dr. McKellops, there would seem to be no necessity for sacrifice. I am sorry that he has returned home and is not present to tell us more about it. If a portion of the root be amputated and patience be exercised, the tooth should be saved. In my own limited experience I have a number of times seen the root denuded, and after amputation the case got well. It is sometimes possible to distend a fistula by means of sea-tangle tent, or compressed sponge, until access can be obtained to the root.

*President Gilmer*—Drainage is sometimes a very important matter.

*Dr. Ames*—The braided horse-hair tube is the best, as the tissues do not grow into it and stop it up.

Dr. Brophy exhibited photographs of a small patient whose features were eaten away in a horrible manner by syphilis, the inoculation having been from the pipe of an old sailor, which was borrowed for the purpose of blowing soap bubbles. The hard palate, the turbinated and the nasal bones, much of the nose, both lips and most of the teeth were gone. There was ectropion of three-fourths of an inch. The boy is improving under treatment, and in time Dr. Brophy hopes, by means of a plastic operation, to naturally amelioriate the condition.

THE RETENTION OF DEAD TEETH IN THE JAW.

The regular essayist, Dr. Homer Judd, was unable to be present, and Dr. Edmund Noyes, at one day's notice, had prepared a paper. He said that an enormously large number of dead teeth and roots are now saved by the dentists. They are filled, crowned, and bridge-work is built upon them. The time has come when people have an exaggerated opinion of the abilities of the dentist. Some practitioners foster this idea by asserting that they never extract teeth. Every pulpless tooth is in an abnormal condition. The most favorable cases for the retention of dead teeth or roots are when, with a good constitution and good teeth, one of them is devitalized and properly treated and filled. In such a case the cicatrix at the foramen is too small to give trouble. Such a tooth in such a mouth will be thoroughly innocuous.

*The Medical Record* once suggested that physicians might find it necessary to order dentists to remove dead teeth. The physician cannot from his own knowledge designate any such. He does not know the condition or symptoms. The common cause for unsuccessful treatment of dead teeth is imperfect and faulty manipulation, and the difficulty in determining the exact condition without resorting to extraction. A chronic state of pericementitis may result from the last point of the root remaining unfilled, and this in time may make an impression upon the general health. Another difficulty may arise from the calcification of pulps previous to devitalization, and this may prevent the perfect filling of root canals. Another source of irritation may be the naturally sharp point of the root. A long continued abscess may bring about this condition, and there may be rough nodules and calculi. Large pulps, in decomposing, may destroy the dentine to so great an extent as to make the whole tooth a source of irritation.

The first indication of disturbance from a dead tooth is usually at the apex. Pain may be caused in almost any tissue, from reflex disturbances arising from a dead tooth. Eye, ear, stomach, and uterus are especially liable, although it may be that this frequency is only apparent because it is more easily recognized. Toothache may also arise from diseased eyes and ears, etc. It is not surprising, then, that eye and ear surgeons are quick to recognize disturbances arising from the teeth, Their observations should be intelligently made, for it is not infrequently the case that some aural disturbance is attributed to a tooth, when extraction does not prove a cure.

In cases of continued alveolar abscess the formation of pus is the most alarming symptom, as it will, in some cases, produce serious drainage to the system. There is much in the constitutional condition which governs the state of dead teeth in the mouth. With some patients abscess is almost certain to supervene when a tooth is devitalized, while with others it rarely occurs. Abscesses sometimes open into the nares or antrum, and retention is incompatible with a state of health. The retention or extraction of dead teeth must be determined like any other surgical operation. The circumstances and probable danger must decide the question.

*Dr. Cushing*—One of the difficulties in the way of saving pulpless teeth is that it is impossible to reach perfection in the filling of roots. Many imperfections escape notice, many are inherent in the tooth, and yet the cases are measurably successful. When we seek for underlying principles, there is little analogy with general surgery. It has been said that we come here to discuss basal laws, and not to relate incidents. All practice is more or less empirical. When it becomes scientific it is because of empirical experimentation. The argument that has been made by medical men would direct that all dead teeth should be extracted for fear of possible bad results, because evil consequences have been observed from the keeping of diseased teeth in the mouth. Such arguments simply point out the ignorance of medical men as to the possibility of treating diseased teeth and restoring them to healthfulness. The instances of the obliteration of tooth-pulps, which the essayist cited, are strong arguments in favor of the possibility of keeping dead teeth, provided they are in an aseptic condition. We must rely upon the tendency of the body to tolerate abnormal conditions. General surgery is founded upon this toleration and adaptability of the human tissues. There are depraved conditions under which the simplest lesions become fatal, but because this is the case we are by no means at liberty to conclude that no surgical operations are warranted. Even though some dead teeth are provocative of trouble, it does not follow that dentists are never justified in treating and filling teeth with devitalized pulps.

*Dr. Barrett*—When a tooth is said to be dead, there is really but one of its tissues that needs protection. The enamel is practically without nourishment. The cementum has still its source of nutriment in the living periosteum. The dentine only is deprived of its normal connection. But even that tissue is isolated and cut off from all other outside tissues, being only in relation with the inert enamel and the living cementum. Its inner surface alone, that towards the pulp, is in its new relation cut off from its normal connection, and it is only from this pulp surface that it is liable to attack. If this face of the dentine be fortified against septic conditions, there can be no ill effects from that dead tooth. How may this be done? By removing all fermentable matter, rendering the cavity and its parietes entirely aseptic, and thus hermetically sealing this cavity against the intrusion of ferment or putrefactive germs or organisms. Make the cavity aseptic by the use of deodorants, disinfectants, germicides or antiseptics, which ever may be demanded, and then seal the foraminal opening with a material that shall be perfectly adaptable to all the inequalities, bland in its character, entirely neutral chemically, a simple substance rather than a complex compound that may possibly be decomposed, non-metallic as being least affected by thermal changes, and one that will be readily tolerated by the tissues. These varying qualities are best found in one of the natural gums—a hydro-carbon rather than a resinous gum—and of all these gutta-perch seems the best adapted. Filling the pulp canals with this material then, and the external cavity with a simple metal like gold or tin, it would seem that the most essential conditions had been met, and such a tooth in a healthy mouth should remain for an indefinite time entirely innocuous.

The society proceeded to the annual election of officers, with the following result:

*President*—W. T. Magill, Rock Island.
*Vice-President*—C. B. Rohland, Alton.
*Secretary*—J. W. Wassall, Chicago.
*Assistant*—Louis Ottofy, Chicago.
*Treasurer*—T. W. Pritchard, White Hall.
*Librarian*—W. B. Ames, Chicago.

*Executive Committee* { E. J. Green, Peoria.

W. H. Taggart, Freeport.

P. J. Kester, Chicago. }

*Board of Examiners* { Garrett Newkirk, Chicago.

E. D. Swain, Chicago.

G. D. Sitherwood, Bloomington. }

Jacksonville was selected as the next place of meeting. The regular bills were ordered paid as follows: Ordinary expense, about $75.00; Secretary's salary, $100.00; Assistant Secretary, $25.00; State Board of Examiners, $200.00; Stenographer, $125.00. The society then adjourned to meet in Jacksonville, the second Tuesday in May, 1887.

